# Significance of depth of invasion determined by MRI in cT1N0 tongue squamous cell carcinoma

**DOI:** 10.1038/s41598-020-61474-5

**Published:** 2020-03-13

**Authors:** Chunmiao Xu, Junhui Yuan, Liuqing Kang, Xiaoxian Zhang, Lifeng Wang, Xuejun Chen, Qi Yao, Hailiang Li

**Affiliations:** Department of Radiology, Affiliated Cancer Hospital of Zhengzhou University, Henan Cancer Hospital, Zhengzhou, Henan P.R. China

**Keywords:** Oral cancer detection, Cancer imaging, Oral cancer detection, Surgical oncology, Risk factors

## Abstract

Depth of invasion (DOI) can be calculated preoperatively by MRI, and whether MRI-determined DOI can predict prognosis as well as whether it can be used as an indicator of neck dissection in cT1N0 tongue squamous cell carcinoma (SCC) remains unknown. The main goal of the current study was to answer these unknowns. A total of 151 patients with surgically treated cT1N0 tongue SCC were retrospectively enrolled, and MRI-determined DOI was measured based on T1-weighted layers with a 3.0T scan. The Chi-square test was used to evaluate the association between clinical pathologic variables and neck lymph node metastasis, and the factors that were significant in the Chi-square test were then analyzed in a multivariate logistic regression analysis model to determine the independent predictors. The main study endpoints were locoregional control (LRC) and disease-specific survival (DSS), and the Kaplan-Meier method (log-rank test) was used to calculate the LRC and DSS rates. The factors that were significant in univariate analysis were then analyzed in the Cox model to determine the independent prognostic factors. A value of p < 0.05 was considered significant, and all statistical analyses were performed with SPSS 20.0. Occult neck lymph node metastasis was noted in 26 (17.2%) patients, and the ROC curve indicated that the optimal cutoff value of MRI-determined DOI was 7.5 mm for predicting neck lymph node metastasis, with a sensitivity of 86.9%. The factors of lymphovascular invasion, MRI-determined DOI, pathologic DOI, and pathologic tumor grade were significantly associated with the presence of neck lymph node metastasis in univariate analysis, and further logistic regression analysis confirmed the independence of lymphovascular invasion, MRI-determined DOI, and pathologic DOI in predicting neck lymph node metastasis. The 5-year LRC and DSS rates were 84% and 90%, respectively. Cox model analysis suggested the MRI-determined DOI was an independent prognostic factor for both LRC and DSS. Therefore, elective neck dissection is suggested if MRI-determined DOI is greater than 7.5 mm in cT1N0 tongue SCC, and MRI-determined DOI ≥ 7.5 mm indicates additional risk for disease recurrence and cancer-related death.

## Introduction

Tongue squamous cell carcinoma (SCC) is the most common malignancy in the oral cavity, and despite advances in diagnosis and treatment, its prognosis has not significantly improved. Locoregional recurrence is the most frequent failure pattern within 2 years after treatment^1^. Increasing evidence indicates that neck lymph node metastasis is one of the most important prognostic factors in tongue SCC^1^, but unfortunately, these positive lymph nodes are usually occult or subclinical at the initial treatment in early-stage tongue SCC. Owing to a wide range of occult metastasis rates^2,3^, either elective neck dissection (END) or the watchful waiting policy has been the favored treatment for cT1N0 tongue SCC^4,5^. Investigators favoring END have commented that it allows accurate disease staging and decision making for the need for adjuvant therapies, and the resection of metastatic lymph nodes can potentially reduce the recurrence risk^6,7^; however, the main concern according to the traditional watchful waiting policy is the associated surgical complications, which include shoulder dysfunction and over-treatment, in patients with no pathologic metastases^8^. Considering that there is no accurate diagnostic procedure for staging the neck preoperatively, elective management of the neck in cT1N0 tongue SCC has been the subject of much debate during the past 3 decades and continues to be controversial.

Depth of invasion (DOI) has now been added in the newest edition of the AJCC tumor-node-metastasis staging system^9^, and abundant literature has shown a significant relationship between DOI and neck lymph node metastasis^10–12^; however, data regarding pathologic DOI usually cannot be obtained by frozen section or incisional biopsy, and DOI might have a limited role in benefiting decision making regarding neck treatment preoperatively. There are various radiology methods, including CT, MRI, PET-CT, and PET-MRI, available for clinical evaluation, but MRI is the most widely used to evaluate soft tissue disease, and current evidence has reported the reliability of MRI in measuring DOI^13,14^ as well as the prognostic value of MRI-determined tumor thickness in tongue SCC^15,16^. MRI-determined DOI is significantly different from MRI-determined tumor thickness, but whether MRI-determined DOI has the same effect as MRI-determined tumor thickness and whether it can be used as an indicator of END in cT1N0 tongue SCC remain unknown; therefore, the main goal of the current study was to clarify these unknowns.

## Results

### Demographic and pathologic data

There were 151 patients (111 males and 40 females) enrolled in total, and the mean age was 57.1 (range: 30–78) years. There were 102 (67.5%) smokers and 61 (40.4%) drinkers. Flap reconstruction was performed in 32 (21.2%) patients: 25 submental island flaps and 7 radial forearm flaps. Perineural invasion and lymphovascular invasion were present in 23 (15.2%) and 19 (12.6%) patients, respectively. Pathologic tumor grades were characterized as low in 75 patients, intermediate in 51 patients, and high in 25 patients. Negative margins were achieved in all patients. Tumor growth patterns of ulcer type, invasive type, and exogenous type were noted in 72 (47.7%), 20 (13.2%), and 59 (39.1%) patients, respectively.

### Cervical metastasis data

Positive neck lymph nodes were noted in 26 (17.2%) patients; there was one positive lymph node in 15 patients, two positive lymph nodes in 7 patients, and three positive lymph nodes in 4 patients. There was no extracapsular spread in any of the positive lymph nodes.

### Image analysis and DOI data

**Figure 1:**
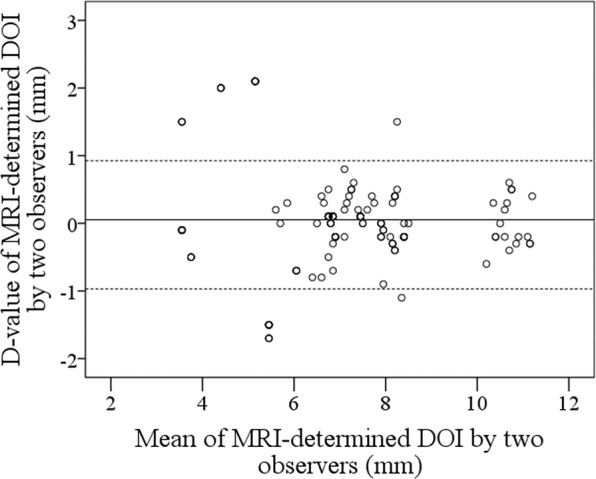
Bland-Altman plots comparing the interobserver variation (ICC = 0.934).

**Figure 2:**
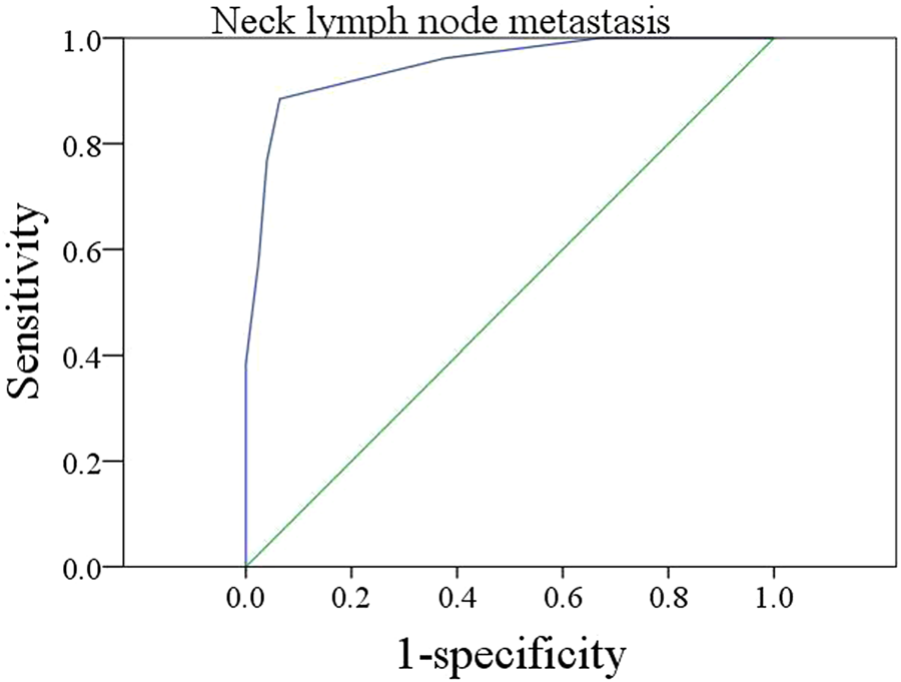
ROC analysis of the optimal cutoff value of MRI-determined DOI for predicting neck lymph node metastasis.

At the time of MRI-determined DOI calculation, two radiologists were blinded to each other; if the measurement results were not consistent, the two observers would discuss and solve the divergence together. Figure 1 depicts the interobserver variation, and the interobserver reliability was excellent (ICC = 0.934). The mean MRI-determined and pathologic DOIs were 6.9 (range: 2–13) mm and 4.2 (range: 1.0–10.0) mm, and the difference was significant (p < 0.001). ROC analysis revealed that the optimal cutoff value of MRI-determined DOI was 7.5 mm for predicting neck lymph node metastasis, with the area under the curve being 0.848; specificity: 82.0%; and sensitivity: 86.9% (Fig. 2).

### Predictors for occult cervical metastasis

As described in Table 1, the factors of lymphovascular invasion (p = 0.015), MRI-determined DOI (p = 0.007), pathologic DOI (p = 0.008), and pathologic tumor grade (p = 0.034) were significantly associated with the presence of neck lymph node metastasis. Further logistic regression analysis confirmed the independence of lymphovascular invasion (p = 0.022, 2.475 [1.233–4.997]), MRI-determined DOI (p = 0.009, 2.978 [1.574–7.332]), and pathologic DOI (p < 0.001, 3.112 [1.812–9.668]) in predicting neck lymph node metastasis.

**Table 1:** Univariate and multivariate analysis of predictors for neck lymph node metastasis.

Variables	Neck lymph node metastasis		Univariate	Logistic regression	
	Positive	Negative	p	p	OR [95% CI]
Age					
≥57	16	74			
<57	10	51	0.825		
Sex					
Male	20	91			
Female	6	34	0.665		
Smokers					
Yes	18	84			
No	8	41	0.841		
Drinkers					
Yes	11	50			
No	15	75	0.827		
Perineural invasion					
Yes	6	17			
No	20	108	0.221		
Lymphovascular invasion					
Yes	7	12			
No	19	113	0.015	0.022	2.475 [1.233–4.997]
Pathologic tumor grade					
Low	8	67			
Intermediate + high	18	58	0.034	0.110	2.414 [0.894–6.114]
MRI-determined DOI^*^					
≥7.5 mm	12	26			
<7.5 mm	14	99	0.007	0.009	2.978 [1.574–7.332]
Pathologic DOI					
>5.0 mm	13	30			
≤5.0 mm	13	95	0.008	<0.001	3.112 [1.812–9.668]
Tumor growth pattern					
Ulcer type	12	60			
Invasive type	5	15			
Exogenous type	9	50	0.599		

*DOI: depth of invasion.

### Survival data

During our follow-up with a mean time of 70.4 (range: 8–103) months, 30 patients underwent adjuvant radiotherapy, and 6 patients also underwent adjuvant chemotherapy. Locoregional recurrence occurred in 22 patients, of whom 16 patients had cervical metastasis at the time of initial treatment, and disease-related death occurred in 13 patients.

**Figure 3:**
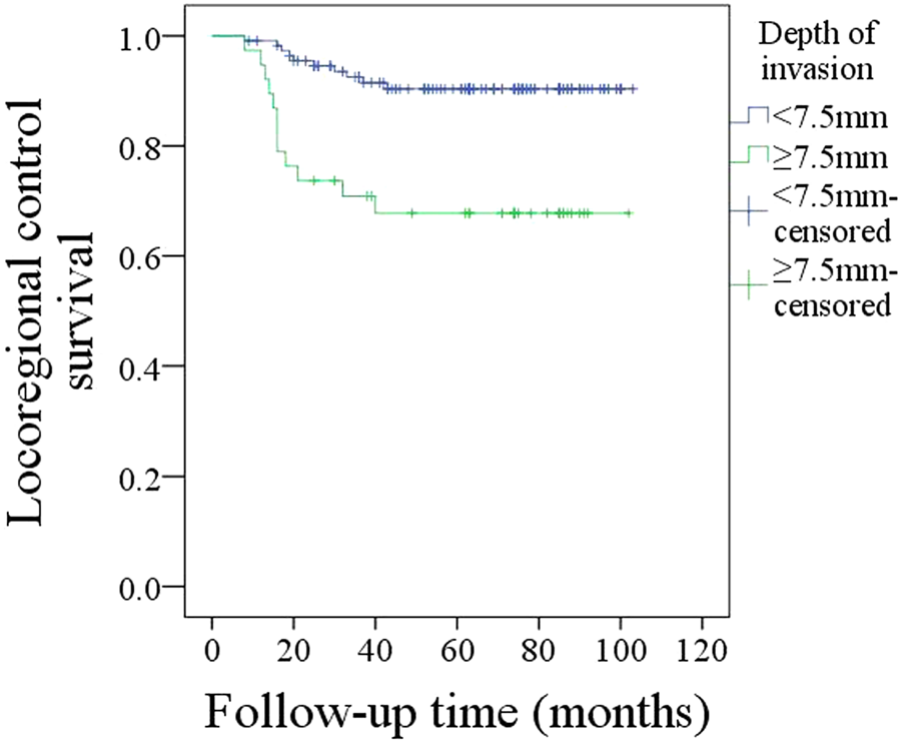
Comparison of locoregional control survival in patients with different MRI-determined depths of invasion (p < 0.001).

**Figure 4:**
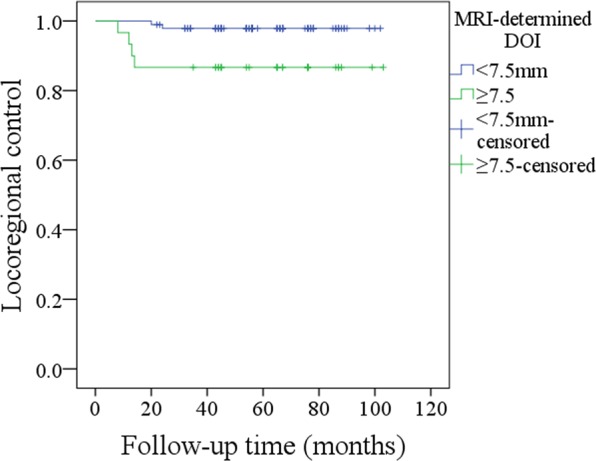
Comparison of locoregional control survival in pN0 patients with different MRI-determined depths of invasion (p = 0.01).

The 5-year LRC rate was 84%. With regard to the prognostic factors for LRC, as described in Table 2, the factors of perineural invasion (p = 0.016), lymphovascular invasion (p = 0.009), MRI-determined DOI (p < 0.001), pathologic DOI (p < 0.001), and neck lymph node metastasis (p = 0.004) were significantly related to LRC. Further, the Cox model indicated the independence of lymphovascular invasion (p = 0.016, 2.007 [1.274–5.732]), MRI-determined DOI (p < 0.001, 2.842 [1.449–7.264]), neck lymph node metastasis (p = 0.035, 1.745 [1.152–4.221]) and pathologic DOI (p < 0.001, 3.246 [1.679–8.336]) in predicting LRC. In patients with MRI-determined DOI ≥ 7.5 mm, the 5-year LRC rate was 68%; in patients with MRI-determined DOI < 7.5 mm, the 5-year LRC rate was 90%, and the difference was significant (Fig. 3, p < 0.001). In the further subgroup analysis of 125 pN0 patients, in 30 patients with MRI-determined DOI ≥ 7.5 mm, the 5-year LRC rate was 87%; in 95 patients with MRI-determined DOI < 7.5 mm, the 5-year LRC rate was 98%, and the difference was significant (Fig. 4, p = 0.01).

**Table 2:** Prognostic factors for the locoregional control survival in patients with T1 tumors.

Variables	Univariate	Cox model	
	Log-rank test	p	RR [95% CI]
Age	0.634		
Sex	0.187		
Smokers	0.334		
Drinkers	0.227		
Neck lymph node metastasis	0.004	0.035	1.745 [1.152–4.221]
Perineural invasion	0.016	0.114	
Lymphovascular invasion	0.009	0.016	2.007 [1.274–5.732]
Pathologic tumor grade	0.095		
MRI-determined DOI	<0.001	<0.001	2.842 [1.449–7.264]
Pathologic DOI	<0.001	<0.001	3.246 [1.679–8.336]
Tumor growth pattern	0.397		
Adjuvant treatment	0.572

**Figure 5:**
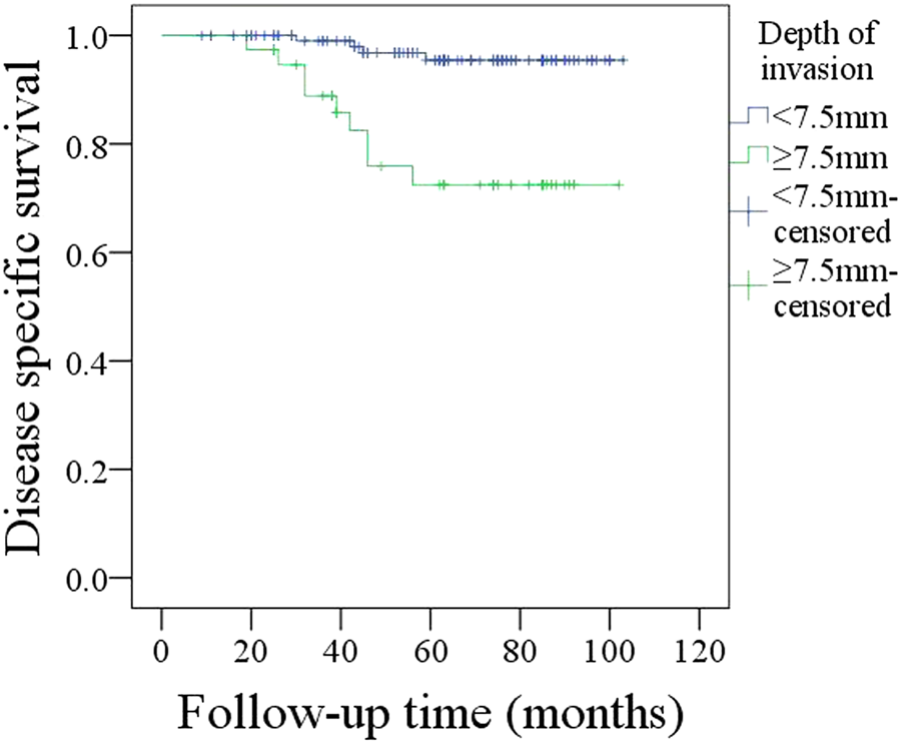
Comparison of disease-specific survival in patients with different MRI-determined depths of invasion (p < 0.001).

The 5-year DSS rate was 90%. With regard to the prognostic factors for DSS, as described in Table 3, the factors of MRI-determined DOI (p < 0.001), pathologic DOI (p < 0.001), and neck lymph node metastasis (p = 0.008) were significantly related to LRC. Further, the Cox model indicated the independence of MRI-determined DOI (p < 0.001, 2.441 [1.635–5.994]), pathologic DOI (p < 0.001, 3.002 [1.753–6.885]), and neck lymph node metastasis (p = 0.005, 2.665 [1.442–5.322]) in predicting DSS. In patients with MRI-determined DOI ≥ 7.5 mm, the 5-year DSS rate was 73%; in patients with MRI-determined DOI < 7.5 mm, the 5-year DSS rate was 96%, and the difference was significant (Fig. 5, p < 0.001).

**Table 3:** Prognostic factors for the disease-specific survival in patients with T1 tumors.

Variables	Univariate	Cox model	
	Log-rank test	p	RR [95% CI]
Age	0.241		
Sex	0.387		
Smokers	0.841		
Drinkers	0.458		
Neck lymph node metastasis	0.008	0.005	2.665 [1.442–5.322]
Perineural invasion	0.089		
Lymphovascular invasion	0.110		
Pathologic tumor grade	0.175		
MRI-determined DOI	<0.001	<0.001	2.441 [1.635–5.994]
Pathologic DOI	<0.001	<0.001	3.002 [1.753–6.885]
Tumor growth pattern	0.422		
Adjuvant treatment	0.631

## Discussion

The most valuable finding in the current study was that MRI-determined DOI was significantly associated with the presence of neck lymph node metastasis, which added a nearly 3-fold risk of neck lymph node metastasis if MRI-determined DOI was greater than 7.5 mm, and MRI-determined DOI was an independent prognostic factor for LRC and DSS. This finding might provide preoperative benefits for neck management in cT1N0 tongue SCC, and neck dissection is strongly suggested if preoperative MRI-determined DOI is greater than 7.5 mm.

The feasibility of measuring DOI by MRI has been widely analyzed^13,14,16,17^. Murakami *et al*.^13^ compared the interrater reliability of different methods of DOI measurement by MRI and found that the axial invasive portion method had excellent interrater reliability. The data in the current study were also obtained with the axial invasive portion method. Lam *et al*.^18^ described that the tumor thickness measured on T1-sequence MRI was 0.8 mm greater than that measured in pathological sections but was 2 mm greater on T2 sequences than that measured in pathological sections on average. A similar finding was also noted by Preda *et al*.^19^. T1-weighted images are more accurate than T2-weighted images for measuring DOI. DOI in T2-weighted images can be overestimated owing to inflammation and surrounding tissue edema. Therefore, in the current study, the MRI-determined DOI was obtained based on T1-weighted sequences to increase our reliability. On the other hand, Park *et al*.^14^ reported that compared to the data measured in postoperative pathological sections, the DOI measured on T1-weighted MRI was 1.5 mm greater, but the mean difference between MRI-determined DOI and pathologic DOI was 2.7 mm in the current study, which is slightly higher than previous findings^14,18,19^. A possible explanation is that only T1 stage tumors were included for analysis, and a relatively high extent of tissue shrinkage exists in small tumors. On the other hand, unlike our study, some of the abovementioned measurements were obtained in contrast-enhanced T1-weighted images, and the use of contrast medium might also affect the reliability of measurements; however, in our cancer center, the primary site of tongue SCC is usually evaluated with nonenhanced MRI.

The presence of neck lymph node metastasis is an important prognostic factor for head and neck SCC^1,3^. END is usually an important component of the primary operation, but owing to the wide range of occult metastasis rates in cT1N0 tongue SCC^7^, the neck management of cT1N0 tongue SCC has been debated over the years and remains controversial. The ideal treatment for patients with cT1N0 tongue SCC must strike a balance between the possible surgical morbidity and optimal oncological outcomes. The common principle is that if the rate of occult metastasis is greater than 20%, cN0 neck nodes should be treated^11,20^. In the current study, the overall occult metastasis rate was 17.2%, but all patients underwent END. There are at least three aspects for the explanation of this phenomenon. First, the high requirement of routine follow-up for the wait-and-see policy was usually not achievable by our patients, as described by our previous studies^21,22^. Patients in our cancer hospital usually come from low-income families and remote districts. Second, there is abundant evidence indicating that patients who do not undergo prophylactic therapy of the clinically N0 neck nodes usually have a low salvage rate of disease recurrence^2–5^. Third, as the most important factor, there are no reliable predictors for occult neck lymph node metastasis from previous studies.

A number of researchers have aimed to explore the potential predictors of occult neck lymph node metastasis. Tumor budding has been defined as the presence of small clusters of cancer cells or isolated single cancer cells, suggesting a highly aggressive biologic behavior and a great possibility of migrating to the adjacent stroma. Xie *et al*.^23^ described that tumor budding intensity was significantly associated with occult lymph node metastasis. Tumor cell proliferation, microvascular regeneration, and tumor metastasis can be promoted by a systemic inflammatory response. Furthermore, the neutrophil-to-lymphocyte ratio (NLR) in peripheral blood is an accurate and reliable inflammatory marker. A high NLR has been significantly related to worse survival in many kinds of solid cancers^24^. Abbate *et al*.^25^ first demonstrated a higher risk for occult neck lymph node metastasis when the pretreatment NLR was greater than 2.93. Loganathan *et al*.^26^ recently reported that END should be considered when the tumor thickness exceeds 5 mm based on the significant relationship between tumor thickness and occult neck lymph node metastasis. Other analyzed variables included perineural invasion, lymphovascular invasion, and pathologic DOI^27,28^. However, data regarding pathologic factors usually cannot be obtained preoperatively, and owing to being easily influenced by infection or inflammation, the pretreatment NLR is nonspecific. Therefore, additional accurate indicators are needed.

As discussed above, MRI-determined DOI can be reliably calculated preoperatively, and our results demonstrated high predictive value of MRI-determined DOI ≥ 7.5 mm for identifying occult metastasis with a sensitivity of 86.9%. In another study by Jung *et al*.^29^, the authors noted that there was a significant association between nodal metastasis and MRI-determined DOI with a cutoff value of 10.5 mm in T1-weighted images, but the authors failed to provide information about the sensitivity, and the variation from our results could be explained by the different calculation methods used for the cutoff value. The potential mechanism for our interesting finding is presented as follows: MRI-determined DOI can indirectly reflect pathologic DOI, as the mean difference between MRI-determined DOI and pathologic DOI was 2.7 mm in the current study; therefore, an MRI-determined DOI cutoff value of 7.5 mm would indicate a pathologic DOI cutoff value of 5.0 mm. Extensive studies have reported that the neck lymph node metastasis risk increases apparently with pathologic DOI > 5.0 mm^10,11,27,28^.

Prognostic factors for tongue SCC have been extensively analyzed, and the widely accepted risk factors include disease stage, tumor differentiation, perineural invasion, lymphovascular invasion, neck lymph node status, pathologic DOI, and so on^1,12,16,22,26,30^. Similar findings were also noted in the current study. However, the significance of MRI-determined DOI in the survival of tongue SCC remains unknown. This is the first study to describe a significant association between MRI-determined DOI and prognosis. MRI-determined DOI ≥ 7.5 mm indicates a higher risk of disease recurrence and cancer-related death. The potential mechanism might be explained by the fact that greater MRI-determined DOI indicates greater pathologic DOI, and the negative effect of pathologic DOI on prognosis has been widely suggested. Tam *et al*.^12^ recently reported that DOI was an independent predictor for both overall survival and DSS. Similar findings were also presented by Iida *et al*.^31^ and Jung *et al*.^29^.

Almost all the studies regarding MRI-determined DOI are focused on evaluating the association between MRI-determined DOI and pathologic DOI. We hope our study will benefit neck management in patients with cT1N0 tongue SCC and help to identify additional methods to improve its progression.

There were some limitations in the current study: the statistical power was decreased by the inherent bias in a retrospective study, we had a small sample size, and additional large randomized control trials are needed to clarify the question.

In summary, there is a significant relationship between MRI-determined DOI and occult neck lymph node metastasis in cT1N0 tongue SCC, and elective neck dissection and adjuvant therapy are suggested if MRI-determined DOI is greater than 7.5 mm; MRI-determined DOI ≥ 7.5 mm indicates additional risk for disease recurrence and cancer-related death.

## Materials and methods

### Ethical consideration

Our study was approved by the Zhengzhou University institutional research committee. Written consent for medical research was obtained from all patients at the time of initial treatment. All procedures were in accordance with the ethical standards of the institutional and/or national research committee and with the 1964 Helsinki declaration and its later amendments or comparable ethical standards.

### Patient selection and data collection

From January 2010 to December 2016, the medical records of adult (≥18 years old) patients with surgically treated tongue SCC were reviewed. The enrolled patients had to meet the following criteria: the disease was primary; the disease was re-staged as cT1N0M0 according to the 7^th^ AJCC classification followed by ultrasound, CT, and MRI examinations; data regarding MRI could be obtained; and data regarding the follow-up could be obtained. Information including age, sex, tumor growth pattern, adverse pathologic characteristics, and follow-up of the enrolled patients was extracted and analyzed. Drinkers were defined as those who consumed at least one alcoholic drink per day for at least 1 year, and smokers were defined as those who smoked on a daily basis or had quit smoking for less than 5 years^1^.

**Figure 6:**
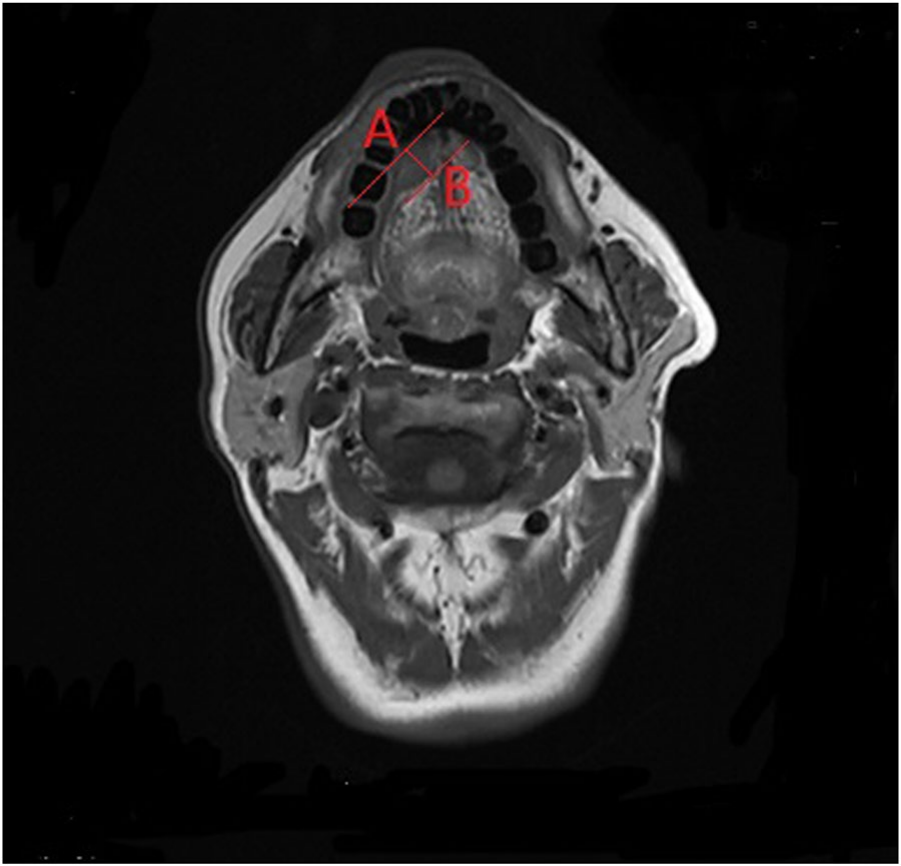
Measurement of the MRI-determined depth of invasion (distance between A and B points) based on the adjacent normal mucosal junction to the deepest infiltration point.

MRI-determined DOI was measured based on T1-weighted layers with a 3.0T scan ^13,14^ it was defined as the vertical distance between the deepest point of tumor infiltration and the simulated normal mucosal junction (Fig. 6). For exogenous tumors, the part above the mucosal surface was neglected, and for ulcerative tumors, the invaginated part was added^17^. The MRI-determined DOI was measured by at least two radiologists with 10 years of working experience.

All pathologic sections were re-reviewed by at least two pathologists, and perineural invasion was considered to be present if tumor cells were identified within the perineural space and/or nerve bundle; lymphovascular infiltration was positive if tumor cells were noted within the lymphovascular channels^32^. The pathologic DOI was measured from the level of the adjacent normal mucosa to the deepest point of tumor infiltration, regardless of the presence or absence of ulceration^9^.

### MRI examination

MRI scanning (SIEMENS Prasma, 3.0T) was performed by several technicians within one week before surgery. The scanning protocol included T1 (TR: 697 ms, TE: 7.1 ms) and T2 (TR: 4070 ms, TE: 112 ms) axial, coronal, and sagittal sequences along with T2 axial and coronal sequences with fat suppression (FS) (TR: 4400 ms, TE: 112 ms). No contrast medium was used during the MRI scan. The images were reconstructed with the thickness of a 1.0-mm slice.

### Treatment principle

In our cancer center, END (levels I–III) was routinely performed in tongue SCC patients with the exception of those with very early-stage disease. Primary tumor excision was usually performed without lip splitting, and the mouth floor tissue was usually preserved unless lingual lymph node metastasis was reported by frozen section. After therapy, the patients were examined every 3 months during the first year, every 6 months during the second year, and once per year after the second year^1^. Once there was suspicion of disease recurrence, aspiration biopsy or incisional biopsy combined with other examinations was performed.

### Data analysis

The MRI-determined and pathologic DOIs were compared using paired Student’s t tests. The ROC curve was used to determine the optimal cutoff of the MRI-determined DOI for predicting neck lymph node metastasis. The interobserver reliability was compared by Bland-Altman plots. The intraclass correlation coefficients (ICCs) were analyzed. An ICC value <0.4 indicated poor agreement, a value varying from 0.4 to 0.75 indicated fair to good agreement, and a value greater than 0.75 indicated excellent agreement. The Chi-square test was used to evaluate the association between clinical pathologic variables and neck lymph node metastasis, and the factors that were significant in the Chi-square test were then analyzed in a multivariate logistic regression analysis model to determine the independent predictors. The primary outcomes were locoregional control (LRC) and disease-specific survival (DSS). The survival time of LRC was calculated from the date of surgery to the date of local or regional recurrence or the last follow-up, and the survival time of DSS was calculated from the date of surgery to the date of cancer-related death or the last follow-up. The Kaplan-Meier method (log-rank test) was used to calculate the LRC and DSS rates. The factors that were significant in univariate analysis were then analyzed in the Cox model to determine the independent prognostic factors. A value of p < 0.05 was considered significant, and all statistical analyses were performed with SPSS 20.0.

## Supplementary information

Supplementary information.

## Data Availability

All data generated or analyzed during this study are included in this published article. The primary data can be obtained from the corresponding author.
