# Supplementary material for: Significance of depth of invasion determined by MRI in cT1N0 tongue squamous cell carcinoma
Source: Sci Rep. 2020 Mar 13;10:4695. doi: 10.1038/s41598-020-61474-5 (PMC7070144; doi:10.1038/s41598-020-61474-5)
Supplement: Supplementary file 1 — Supplementary information. [file 41598_2020_61474_MOESM1_ESM.pdf]

Significance of depth of invasion determined by MRI in cT1N0 tongue squamous cell carcinoma

Chunmiao Xu [1,#], Junhui Yuan [1,#], Liuqing Kang [1], Xiaoxian Zhang [1], Lifeng Wang [1]
Xuejun Chen[1], Qi Yao [1], Hailiang Li [1,*]

1 Department of Radiology, Affiliated Cancer Hospital of Zhengzhou University, Henan Cancer Hospital, Zhengzhou, Henan, PR China

#: The first two authors make the same contribution

*: Corresponding author at: Department of Radiology, Affiliated Cancer Hospital of Zhengzhou University, Henan Cancer Hospital, Zhengzhou, Henan, PR China. Tel: 37165588452; Fax: 8665587222; Email: xuchunmiao456@sina.com

Supply table: Tumor stage change according to the different classification system

Tumor stage	7th AJCC classification	8th AJCC classification
T1	151	111
T2	0	40
